# Let’s talk about body neutrality: content analysis of #bodyneutrality on TikTok

**DOI:** 10.1186/s40337-024-01163-0

**Published:** 2024-12-04

**Authors:** Paolo Mancin, Helena Vall-Roqué, Wesley Grey, Scott Griffiths, Sarah Bonell

**Affiliations:** 1 Department of General Psychology, University of Padua, Via Venezia, 8, 35131 Padua, Italy; 2 Department of Clinical Psychology and Psychobiology, Faculty of Psychology, University of Barcelona, Barcelona, Spain; 3 Melbourne School of Psychological Sciences, University of Melbourne, Melbourne, Australia; 4 Bolton Clarke Research Institute, Melbourne, Australia

**Keywords:** Body neutrality, Body positivity, TikTok, Body image, Social media, Content analysis

## Abstract

**Background:**

To date, over 1.3 billion videos with the hashtag #bodyneutrality have been viewed on TikTok. Despite this, little existing literature has unpacked how body neutrality is conceptualised on TikTok. We examined how TikTok creators construct meaning and generate discourse surrounding #bodyneutrality.

**Methods:**

Extending on previous works in the body neutrality space, we conducted a hybrid content/thematic analysis of TikTok videos in three different languages (English, Spanish, and Italian). Initially, 300 videos displaying “#bodyneutrality” were identified on TikTok. The first 178 TikTok videos were analysed, following the principles of data saturation and feasibility.

**Results:**

We developed three themes: (1) The normalisation of diverse bodies, (2) The rejection of appearance as fundamentally important, and (3) Body neutrality is (better than) body positivity.

**Conclusions:**

In line with conceptualisations of body neutrality in existing literature, some content emphasised the importance of devaluing physical appearance. Building on existing definitions, most creators also framed body neutrality as speaking to the fundamental humanness of owning a body and attempted to normalise various body shapes/sizes. Conversely, some content employed #bodyneutrality to promote or examine body positivity principles or to condemn appearance-based stigmatisation. Our study is one of the first to examine how body neutrality is understood and employed by people in the real world.

## Abstract (plain language summary)

Content promoting body neutrality, often shared with #bodyneutrality, has recently become more popular on social media, especially on the widely-used platform TikTok. Consequently, the current study aimed to examine how body neutrality is represented and discussed on TikTok. After conducting a hybrid content/thematic analysis of 178 TikTok videos in English, Spanish, and Italian, we identified three main themes to describe body neutrality: (1) The normalisation of diverse bodies, (2) The rejection of appearance as fundamentally important, and (3) Body neutrality is (better than) body positivity. In the analysed videos, body neutrality content depicted the experience of having a body as fundamentally normal, natural, and human. Moreover, many videos deemphasized the importance placed on beauty in favour of individual characteristics (e.g., hobbies). Finally, some videos compared body neutrality with body positivity, highlighting similarities and differences between the two.

## Introduction

Body neutrality is a popular culture body image movement that has gained recent attention in the body image field. In contrast with body positivity (which argues that people of all shapes and sizes are beautiful and should love their bodies), body neutrality emphasises that appearance should be devalued altogether. In other words, to be ‘body neutral’ is to reject the idea that appearance – whether good or bad – determines one’s value [1, 2]. Pellizzer and Wade’s seminal work sought to define body neutrality by synthesising online discourse pertaining to the topic [3]. Specifically, they explored body neutrality content across 107 websites (e.g., blogs, news articles) and found that body neutrality is typically characterised by three core elements: (1) adopting a neutral attitude toward the body is more realistic, mindful, and flexible than being body positive, (2) appreciating, respecting, and caring for the functionality of the body is paramount, and (3) self-worth should not be defined by appearance. Overall, body neutrality positions appearance and attractiveness as broadly unimportant and encourages people to instead focus on what the body can do (e.g., the body can help us to partake in hobbies and transport us to new places).

### How social media impacts body image

Body image is a complex psychological construct broadly comprising thoughts, perceptions, emotions, and behaviours related to the body and to the physical appearance [4, 5]. It could be divided in negative and positive body image, which are two different constructs characterized by unique components [6]. Scholars have argued that body image, particularly negative body image, could be influenced by sociocultural influences, as described within the Tripartite Influence Model [7]. According to this model, family, peers, and media – including social media such as Instagram and TikTok – influence body satisfaction and eating behaviours through the internalisation of dysfunctional beauty ideals (e.g., thinness and muscularity) and appearance-focused social comparison [7–9]. Hence, social media use – and, in particular, appearance-focused use (e.g., engaging in photo-based activities or viewing appearance-focused content) – could shape users’ relationships with their bodies. Corroborating this hypothesis, scholars have found associations between different dimensions of social media use and body image and eating behaviours [10–15]. 

Among the different forms of social media content, scholars delved into the effect of body positivity content. Interestingly, extant findings on body positive content are mixed, with positive, detrimental, and negligible effects on body image dimensions reported across existing literature [14, 16–24]. A possible explanation of this mixed findings lays in the misrepresentation of body positivity content on social media, which could often portray content that do not align with its core principles (e.g., thin women, self-objectifying poses) [25–27]. Thus, in recent years, scholars have begun to investigate the effect of body neutrality content. Exposure to its content could reduce negative body image and improve positive body image. Indeed, body neutrality content could reduce the emphasis on appearance, which characterizes negative body image, and promote a respect toward the body and its functionality, which characterizes positive body image [27]. In support of this hypothesis, exposure to body neutral content and messages demonstrated to improve body satisfaction, decrease appearance comparison, and reduce endorsement of sociocultural beauty ideals [28, 29]. Despite these promising results, little is known about how body neutrality is represented on social media; that is, while it seems that body neutrality content has a positive impact on body image, we do not actually know what sorts of themes, messaging, and information this content typically subsumes.

### TikTok and body neutrality

TikTok is fast becoming the most popular and successful social media platform worldwide. The number of TikTok users has increased substantially over the past few years, with approximately 1.7 billion people now using TikTok [30]. TikTok is therefore arguably one of the largest drivers of cultural discourse (and information dissemination) in modern society; the platform enables users to rapidly disseminate ideas and beliefs to billions of people at a time [31, 32]. As of 2023, over 1.3 billion videos with the hashtag #bodyneutrality have been viewed on TikTok. Despite this, only few studies to date have examined body neutrality content on TikTok. Hallward et al. explored and compared the content within #bodypositivity and #bodyneutrality hashtags on TikTok [1]. In doing so, they separately coded body positivity and body neutrality content and developed joint themes that captured content across both hashtags, finding only minor differences in video content between the two. Ultimately, they concluded that content across both hashtags tended to challenge societal beauty standards and attempted to promote positive body image. While Hallward et al.’s study progressed our understanding of body neutrality discourse on TikTok, future research is required [1]. 

Given that social media discourse evolves rapidly (particularly in the body image space, where movements are constantly progressing), it is important that findings would be replicated over time. Data analysed by Hallward et al. were collected on 20th July 2022 [1]. Since then, body neutrality has become far more pervasive and widely adopted as a leading societal body image movement (e.g., media coverage of body neutrality has expanded) [33]. Furthermore, Hallward et al. collected TikTok data solely within Canada and only analysed videos presented in English [1]. Because TikTok’s sophisticated algorithm displays targeted content to users depending on location and language settings, Hallward et al.’s findings may be limited in their generalisability to other countries (especially to non-English First Language countries) [1]. Finally, Hallward et al. analysed body positivity and body neutrality jointly: thus, their analyses could have limited applicability to describe body neutrality per se [1]. 

### The current study

The current study expanded on previous research to examine how body neutrality is constructed on TikTok. We used hybrid content/thematic analysis to examine TikTok video content across three languages (English, Spanish, and Italian) posted with the hashtag #bodyneutrality. Moreover, we included a quantitative analysis of the descriptive features of each video. In doing so, we provided a deeper understanding of how body neutrality is represented online.

## Methodology

### Data availability statement and ethical considerations

In the present study, we only examined TikTok videos that were made publicly accessible and downloadable at the time of the analyses (i.e., to protect creator privacy, we did not examine private videos). The TikTok URLs that were employed for the analysis of this study are available on the Open Science Framework (OSF; https://osf.io/kf8my/?view_only=bd71ad360689487b91ff10bf75146a81*).* This study was approved by the University of Melbourne’s Office of Research Ethics and Integrity (Ethics ID: 26461).

### Materials and procedure

Before conducting our hybrid content/thematic analysis, we quantitatively analysed demographic/descriptive features for content creators within our video data [34]. This approach was adopted based on previous studies that pursued similar objectives [35, 36]. 

#### Collection of the content

The authors created two new TikTok accounts using different devices (iOS and Android) located in Melbourne, Australia, to collect TikTok videos. On December 12th, 2022, The first and the second authors opened the search bar within the TikTok app and searched for “body neutrality”. Then, they entered the section “hashtag” and selected “#bodyneutrality”.

As per existing social media content analysis studies, authors downloaded the first 300 TikTok videos presented for potential inclusion in the present study [1, 26, 35–37]. TikTok videos are not typically shown in chronological order. Instead, content is organized based on a complex algorithm that takes into account several factors: the popularity of the post (determined by views, likes, comments, and shares), the influence of the creator (measured by their followers and engagement levels), past interactions with similar content (offering a personalized experience for users), and the geographic location of the device accessing TikTok. We selected a large number of TikTok videos to provide a reliable starting point for the analyses. The authors collected the URL of each TikTok video and compared them between devices to make sure that the same TikTok videos appeared in the same order regardless of device. To preserve users’ privacy, we analysed only publicly available, downloadable TikTok videos [1, 35]. Videos in English, Spanish, and Italian were included in the analyses. We decided to consider these languages for two main reasons: (1) including content in languages different from English would enrich the generalizability of the findings and (2) the coders were native and/or fluent in these languages. However, unlike with the English videos, authors did not use data saturation to determine how many Spanish and Italian videos to include in the present study. Rather, out of the first videos downloaded for inclusion in the present study, only 28 were by Spanish/Italian content creators. Thus, we were limited by feasibility constraints.

We chose to retain videos with the cross-over hashtag #bodypositivity in our analysis because we were interested in whether and how content creators might speak to the similarities and differences between these two movements, as well as creators’ perceptions of the utility of body neutrality compared to body positivity.

#### Quantitative demographic and descriptive analyses

The authors developed a “quantitative measures guide” to analyse demographic and descriptive data for content creators within the present study. This guide was based on previous content analyses conducted on social media data and was used in place of being able to collect actual demographic data from content creators [25, 26, 35, 38]. Our guide contained measures of the following content creator characteristics: perceived gender presentation, age, ethnicity/race, body exposure, tightness of clothing, and part of the body shown.

While completing the guide, each coder was asked to select an option out of several alternatives for each examined characteristic. The coders were asked to code perceived gender presentation of the content creator (i.e., the main individual featured in the TikTok video) choosing among three options: “masculine”, “feminine”, and “androgynous”. Perceived age was coded considering five clusters: less than 15 years, between 15 and 20 years, between 20 and 29 years, between 30 and 39 years, and between 40 and 49 years. Race/ethnicity was coded as “African-American/Black”, “Asian”, “White/Caucasian”, “Indigenous”, “Latinx”, “Middle Eastern”, or “Other”. The measures for body exposure and clothing tightness were developed based on previous studies [25, 26]. Body exposure had the following options: “Not at all revealing”, “Slightly revealing”, “Moderately revealing”, “Very revealing”, “Extremely revealing”, and “Not shown”. Clothing tightness was coded choosing among “Tight fitting”, “Normal/comfortable fit”, “Loose/baggy fit”, “Swimsuit/Underwear”, and “Naked”. We also developed a measure of the “part of the body shown” and defined several options (“Just face”, “Upper body and face”, “Upper body without face”, “Lower body without face”, “Upper body and lower body without face”, “Whole body”). Shared agreement on the meaning of each measure and how to choose among its options was reached among the coders after a training with TikTok videos that were not included/utilized in the final analyses. Whenever coding a certain characteristic was considered impossible (e.g., videos without human people portrayed), “Not Applicable (N/A)” was utilized.

We also collected information that would specifically refer to TikTok video characteristics: additional hashtags used, video length, and the number of people in the video. This information was recorded on a separate sheet, without previously defined codes. Since Spanish and Italian videos included additional hashtags in their native language, this information was only collected from the 150 English videos.

First, the first and the second authors used the quantitative measures guide to independently assess each video included in the analysis (178 TikTok videos). Second, inter-rater agreement and Cohen’s kappa were calculated to determine the reliability of scoring. The kappa statistic was interpreted following McHugh’s recommendations (> 0.90: almost perfect agreement; 0.80-0.90: strong agreement; 0.60-0.79: moderate agreement; <0.60: inadequate agreement) [39]. Finally, the first and the second authors independently re-watched all the videos that included measurement disagreements and discrepancies were discussed and resolved in several meetings until 100% intercoder agreement was reached for all variables. Inter-rater agreement and Cohen’s kappa are presented in Table 1.

**Table 1:** Initial inter-rater agreement for the descriptive variables

Variable	Interrater Agreement (%)	Cohen’s kappa
Gender presentation	100	1.0
Perceived age	85.6	0.78
Race/ethnicity	95	0.83
Number of people in the video	99.3	0.98
Body exposure	84.2	0.76
Clothing tightness	92.8	0.89
Part of the body shown	97.8	0.96

*Note* These indices reflect the agreements on each demographic and descriptive data as coded after the independent assessment (prior establishing 100% intercoder agreement)

### Qualitative analysis: hybrid content/thematic analysis

#### Step 1: familiarisation with the TikTok videos

In line with Braun and Clarke’s approach to reflexive thematic analysis, data familiarisation was the first step in our analysis plan [34]. The first, the second, and the third authors familiarized themselves with all the videos (watching them no more than two times) and wrote down general comments about them. The aim for this phase was to focus on developing general impressions about the videos.

#### Steps 2, 3, and 4: coding TikTok videos and iterative theme development

As per reflexive thematic analysis, researchers typically do not develop codes or themes until after data collection has ceased [34]. However, in the present study, we chose to adopt an approach to coding and initial theme generation (drawing inspiration from Grounded Theory, codebook thematic analysis, and reflexive thematic analysis). We adopted this method because it aligned with previous qualitative studies that focused on analysing TikTok videos [35]. Specifically, the first author coded the first of our 50 (English) TikTok videos and developed themes pertaining to these videos. After developing initial themes, the first authors consulted the second and the third authors to discuss them. The main aim was not to “reach a consensus” on themes, but to include every opinion as a process of merging ideas [40]. Next, the first author coded an additional 50 videos and amended themes to account for these new data, then once again met with the second and the third authors to discuss. The first author then conducted one more round of this process; he coded an additional 50 videos, adjusted themes, and met with the second and the third authors one last time. In this way, coding, initial theme development, and theme revision were iterative processes in the present study. Following Braun and Clark’s and Herrick et al.’s suggestions, the main objective in coding was to code enough content to reach reasonable ‘data saturation’, wherein analysing subsequent material would not substantively contribute to new codes or themes being developed [34, 35]. Authors determined that after 150 videos had been analysed, data saturation had been met.

#### Step 5: coding of content in Spanish and Italian language and further theme development

The first and the second authors additionally coded 15 TikTok videos in Spanish and 13 TikTok videos in Italian. Codes from the Spanish and Italian videos were used to further refine initial themes generated from the English videos previously analysed in Steps 2–4.

#### Step 6: refinement of themes

Once themes were developed, the first author re-watched all the TikTok videos to refine themes names and make a few small adjustments to codes (e.g., wordings) and quote selection.

### Researcher positionality statement

The authors acknowledge that all stages of the research process were shaped by their prior experiences and knowledge [34]. The first author defines himself as a White cisgender gay man living in an average-size body. Because he identifies as a sexual minority man, he acknowledges that he may have experienced a threat to his body image and the pressure to adhere to stereotypical beauty ideals that are presented within the gay community. Moreover, he acknowledges that he cannot entirely understand the experience of body image issues experienced by people who identify as other genders, such as women. This may have impacted coding the content since he perceived that the majority of TikTok creators displayed a feminine gender presentation. The second author identifies as a cisgender, White, Catalan/Spanish woman, who lives in a thin-size body and has experienced her own struggles with body image. Lastly, the third author is a White gay man who has experienced eating and body image concerns in the past. Similar to the first author, he cannot fully understand the experience of women or feminine people vis-à-vis body image, but understands how social pressure and appearance ideals can be particularly damaging and recognizes the inherent value in body neutral social media content.

### Philosophical assumptions

This study was conducted utilizing contextualist (epistemology) and relativist (ontology) approaches. We assumed that TikTok creators were generating meaning when they spoke about body neutrality and that this meaning differed between users. We did not seek to establish one true meaning for body neutrality; rather, we wanted to know how meaning was uniquely constructed across different creators.

## Results

### Quantitative analysis: descriptive and demographic characteristics

Within the 178 analysed videos, TikTok content mainly depicted individuals who were perceived by the authors as looking ‘feminine’, between 20 and 29 years old, and White/Caucasian. On average, videos showcased at least one person (only 3 videos did not portray a person) and were 60 s long. Most of the videos included several hashtags besides #bodyneutrality. The most frequent were #bodypositivity (43 videos), #bodyneutral (27 videos), #fyp (21 videos), #bodyacceptance (16 videos), #selflove (15 videos), and #normalizenormalbodies (14 videos). The details of the descriptive features of the videos are presented in Table 2.

**Table 2:** Descriptive aspects of the TikTok videos

Variable	Frequency	Relative Frequency (%)
*Gender presentation*		
Feminine	170	95,5
Masculine	4	2,2
Androgynous	1	0,6
N/A	3	1,7
*Perceived age*		
< 15	1	0,6
15–20	57	32,0
20s	84	47,2
30s	31	17,4
40s	2	1,1
N/A	3	1,7
*Race/ethnicity*		
African-American/Black	8	4,5
Asian	2	1,1
White/Caucasian	138	77,5
Indigenous	0	0,0
Latinx	19	10,7
Middle Eastern	0	0,0
Other	8	4,5
N/A	3	1,7
*Body exposure*		
Not at all revealing	71	39,9
Slightly revealing	44	24,7
Moderately revealing	21	11,8
Very revealing	24	13,5
Extremely revealing	5	2,8
Not shown	13	7,3
*Clothing tightness*		
Tight fitting	36	20,2
Normal/comfortable fit	88	49,4
Loose/baggy fit	16	9,0
Swimsuit/Underwear	24	13,5
Naked	0	0,0
N/A	14	7,9
*Part of the body shown*		
Just face	15	8,4
Upper body and face	84	47,2
Upper body without face	0	0,0
Lower body without face	0	0,0
Upper body and lower body without face	0	0,0
Whole body	76	42,7
N/A	3	1,7

*Note* The Relative Frequency denotes the percentage of the videos perceived as displaying each feature after establishing the 100% intercoder agreement

### Qualitative analysis: themes developed

The authors developed three themes that describe how TikTok creators conceptualise and explain body neutrality to their audiences: (1) The normalisation of diverse bodies, (2) The rejection of appearance as fundamentally important, and (3) Body neutrality is (better than) body positivity.

#### Theme 1: the normalisation of diverse bodies

This theme relates to creators’ attempts to dismantle societal discourse surrounding (1) the “right way” to have a body and (2) the “healthy way” to have a body. Unlike body positivity principles, data under this theme did not speak to every body type inherently being beautiful; rather, data were reflective of every body type being valid (i.e., normal, functional, healthy).

Firstly, participants focused on normalising different body sizes, shapes, and changes over time. One TikTok creator (feminine, 20–29 years old) encouraged viewers to normalize people carrying fat on their bellies. While touching and showing their own belly, they acknowledged that it is not ‘flat’ even after “sucking in”. They asked viewers: “can we just show this off and normalize this real’ quick?”. They also added that “a lot of women have this, and some, a lot that don’t even have kids […] and they’re fit and everything”. This TikTok creator sought to legitimise and de-stigmatise their own body and the bodies of those who look like them. Similarly, a Spanish-speaking creator (feminine, 20–29 years old) talked about how Marilyn Monroe’s body has often been perceived as a “plus-sized body” while it was actually a normative body, and discussed how this is harmful: “We have this image of Marilyn Monroe as plus-size, curvy girl… But calling Marilyn Monroe plus-size says a lot about how we see sizes nowadays and the damage this does. […] Marilyn Monroe, even though she was curvy, had a small body. I think I don’t need to say that perpetuating a 36 [a small clothing size] as curvy or plus-size is extremely harmful. […] Let’s showcase people that are actually representing the bodies that what we want to represent. Otherwise, we are just contributing to the lack of body diversity in social media.” (translated).

Other TikTok creators revealed or spoke to parts of their bodies that could be considered socially undesirable, aiming at normalizing them. For example, a TikTok creator (feminine, 20–29 years old) began their TikTok video wanting to remind the viewer(s) that: “being bloated is a very normal, and very human experience. But so is having fat! So, it’s okay if you’re not just bloated, and it’s okay if it’s just fat”. While addressing the viewer, they posed in front of the camera, showcasing their body in different positions. Another interesting example is provided by a TikTok creator (feminine, 15–20 years old) who described an experience they had during a Pilates class. During the class, this creator spotted a woman across the room who appeared to be upset by their body hair. In response to this, the creator ensured viewers that they would once again display their body hair proudly in upcoming pilates lessons. They concluded the video by saying “mind your own body babes”. In this sense, the creator positioned negative body commentary as harmful and framed having body hair as normal and valid. In other TikTok videos, the word “fat” itself was presented by creators as an adjective only (“fat is not a bad word to me, it can be a neutral word”; “fat is a descriptive word”). Fat was framed as neither bad nor beautiful, but rather purely descriptive.

TikTok creators also sought to normalize body changes over time (e.g., ageing and changes after childbirth). For example, one TikTok creator (feminine, 20–29 years old) lip-synced to a pre-recorded sound while projecting text on-screen that spoke to their personal body image experiences after childbirth. During the video, they acknowledged that they never “bounced back” after pregnancies and that they gained weight while breastfeeding. Importantly, they then shared that they are not interested in trying to lose weight and suggested that they do not wish to go back to a “normal” body since they believe “all bodies are normal”.

As well as normalising different ways that bodies might look or change over time (i.e., all bodies as valid), content creators also sought to normalise the idea that all bodies can be healthy. TikTok creators emphasized that health and size are not synonymous (“it [weight] is just a number”), and that “being plus size does not inherently mean that you are unhealthy, just as being thin does not inherently mean that you are healthy” (feminine, 20–29 years old).

In sum, this theme speaks to creators’ attempts to:

- redefine what is meant by a good/healthy body, which does not align with adherence with sociocultural beauty standards;
- normalize different body sizes, shapes, and changes over time, as well as fatness and the presence of perceived flaws;
- frame “having a body” as a fundamentally human experience.

#### Theme 2: the rejection of appearance as fundamentally important

Several videos foregrounded the idea that appearance is not an important component of being a good or happy human being. For example, a creator posted a TikTok video in which they (feminine, 15–20 years old) asked the viewer(s) to join them in saying some affirmations: (1) “my body is the least interesting thing about me”, (2) “I am important simply because I exist”, and (3) “when we are all dust no one will be able to tell whether or not I was hot”.

Sometimes, creators reflected on the alternative avenues (i.e., not appearance) through which they develop self-worth. For example, while asking people to stop commenting on their weight, a TikTok creator (feminine, 20–29 years old) said: “I’ve got to a point in my life where there are a million things I’d rather think about: planting wildflowers in my garden, spending time with my cows, running a business that creates beautiful things”. Likewise, some TikTok Creators fostered the idea that traditionally appearance-focused mediums (e.g., clothing, makeup) had the potential to be ‘re-branded’ in a body neutral way. For example, a creator emphasised that, through body neutrality, clothes become an expression of their personal taste rather than their appearance: “I wear the clothes, the clothes don’t wear me” (feminine, 15–20 years old).

Body neutrality content on TikTok also emphasised the importance of condemning all appearance-related commentary; that is, creators argued that no one should comment on another person’s body, even if comments are intended as compliments. This concept was presented in a video, in which the TikTok creator (feminine, 20–29 years old) asked people to “please stop mentioning or commenting on my weight”. “When I say I don’t wanna mention my weight, […] I’m talking about ever: I’m putting on, if I’m losing it, if it’s saying the same I don’t wanna talk about it. Two main reasons for this. Firstly, people have started greeting me with ‘oh my god you look amazing, have you lost weight?!’ every time I see them. I know they mean well, but do they think I’m not gonna notice when they stop doing that? When they stop telling me I look great? Because they will. Because I will put weight on again”. In this sense, the creator emphasised the importance of de-centralising appearance in social interactions altogether. Similarly, an Italian-speaking creator (feminine, 20–29 years old) explained that “you’ve become thinner!” is not always a compliment. To de-centralise appearance, the creator suggested alternative compliments such as “you have a beautiful aura/light!” (translated).

Creators also spoke to the ways in which body neutrality had actively served to improve their mental health. For example, a creator (feminine, 30–39 years old) noted that “when you’re neutral about the way that you face the world, I feel like you start to like yourself. And once you like yourself, you don’t let people talk crazy to you. The world is your mirror you start to surround yourself with people who fucking like you.” Here, rejecting beauty empowered the creator to form a stronger sense of self and consequently form healthier social relationships.

Finally, some videos framed body functionality as a core proponent of body neutrality and/or as a tool through which to improve body image in general. For example, a creator (feminine, 30–39 years old) outlined that focusing “on the amazing things that [the] body does” could make people “super grateful” for their body regardless of its appearance. Likewise, another creator (feminine, 30–39 years old) showcased their whole body during a TikTok video while playing an audio recording of actress Emma Thompson in the background stating: “Don’t waste your life’s purpose worrying about your body. This is your vessel, it’s your house, it’s where you live. There’s no point in judging it, absolutely no point!”. Here, Emma Thompson describes the role of the body as home/vessel; rather than something fundamentally aesthetic, the body is framed as a means through which we can do or be. In doing so, the audio conveys great appreciation for body functionalities and capabilities. This audio recording was also utilised in several other TikTok videos, suggesting that this messaging resonated with multiple body neutrality content creators.

In sum, this theme refers to TikTok creators attempts to:

- detach one’s sense of self from appearance and discover new means through which to develop self-worth;
- ‘re-brand’ traditional appearance-focused mediums as means to express personal style and taste;
- condemn appearance-oriented comments as unnecessary and irrelevant;
- emphasise the benefits of adopting a ‘body neutral lifestyle’;
- speak to the power of body functionality as a means to shift how we frame our understanding of bodies overall (i.e., bodies as objects versus bodies as tools/vessels).

#### Theme 3: body neutrality is (better than) body positivity

Under this theme, we examine the ways in which creators made sense of body neutrality by presenting it through (or in contrast with) a body positivity framework. The overlap and distinction between body neutrality and body positivity was touched on by some creators but not others, and several creators conflated the two terms. Those that did distinguish between body positivity and body neutrality, however, typically framed body neutrality as more realistic and useful than body positivity.

Firstly, several creators touched on the overlapping principles that underscore both body neutrality and body positivity. Indeed, since they both aim to foster a functional relation with the body, they might share principles described in the larger construct of positive body image. For example, both movements suggest that body insecurities are normal and that feeling self-conscious about our bodies at times is unavoidable in modern society. Accordingly, a creator (feminine, 30–39 years old) noted that someone “can’t feel body confident or love [their] body every single moment of every single day”. They also mentioned that “these thoughts are pretty normal […] in this aesthetically driven world we live in”. Moreover, in line with both body positivity and body neutrality, celebration and respect for body functionalities is a shared feature. Indeed, as previously stated, creators also spoke to the mental health benefits of joyful movement (as opposed to exercising to lose weight). For example, a creator (feminine, 20–29 years old) emphasised that “fitness, health, movement, and exercise have so many more benefits and purposes other than shrinking a number on a scale”.

Likewise, another TikTok creator (feminine, 20–29 years old) shared their body image experiences (“my body dysmorphia”) with viewers by speaking to several pictures of themself that they displayed in the background of the video. At the beginning of the TikTok video, they addressed viewer(s) by acknowledging they had recently gained weight. While showing pictures of before the weight gain and describing their dysfunctional body-oriented behaviours (e.g., restrictive eating, excessive exercising, and monitor of the weight), they said that they were “miserable” but “in a small body, so I was ‘happy’”. Towards the end of the video, they showed a recent photo of themself and explained that they “gained weight as a result of focusing on my mental health, my emotional health, and just taking care of myself”. They also admitted that “this is the happiest and most confident version of myself”. They concluded by saying: “you have to go on that journey and do that work outside of just losing weight because being validated by a number is not enough”. In this sense, the creator framed the self as greater than a ‘number on the scale’ (in line with existing body neutrality online discourse), which entails disengaging from appearance to validate self-worth. However, they suggested they are most confident now that they’re at a higher, more sustainable weight, showcasing appreciation for their current body (in line with body positivity).

Secondly, some creators spoke to the underlying differences between body neutrality and body positivity. A creator (feminine, 30–39 years old) communicated to the viewer that: “practicing body neutrality [is] thinking: okay, this is what my body looks like. Not “YES I LOVE my POOCH. It’s amazing! Nothing wrong with that thinking [body positivity], but it’s not what I’m focusing on”. Another creator (feminine, 20–29 years old) similarly produced a video in which they explained that “unlike body positivity, that focuses on loving your body and the way it looks, body neutrality focuses on the non-physical aspects of yourself and doesn’t even focus on your appearance at all”. Interestingly, this creator went on to frame body neutrality as (in some ways) superior to body positivity. They commented: “sometimes I feel so much pressure to feel hot and sexy when in reality I even don’t want to think about my appearance” and suggest that “your worth should have nothing to do with your appearance”. Here, body neutrality (unlike body positivity) does not demand that the individual feel confident in their appearance; rather, the individual need only to move away from focusing on appearance altogether.

Similarly, a pair of Spanish-speaking content creators (feminine, 20–29 years old) discussed body neutrality as fundamentally different from body positivity. These creators outlined that “the point of body neutrality is: I know that my body is not pretty, but I have a neutral relationship with it. I cannot love it because I was told that I had to hate it, but I’m going to start unlearning this.” They also described how they prefer body neutrality to body positivity “because it’s like a more honest movement, it’s not like ‘oh, we are beautiful’ like body positivity. […]”.

Finally, some content creators on TikTok using the hashtag #bodyneutrality seem to present video content more aligned with body positivity principles, such as praising the appearance of bodies that do not align with sociocultural beauty ideals. For example, a creator (feminine, 15–20 years old) posted a video in which they were dancing under text that read “ugh my belly, it’s so cute”. Another example is provided by a creator (feminine, 15–20 years old) who told viewers that they had often been bigger than their romantic partners (“I’ve been five ten, taller than most men, and a hefty weight my whole adult life”), but that this never stopped them from getting attention from “short kings”. In these videos, beauty was presented as fundamentally valuable but achievable for all different kinds of bodies. This framing does not align with established online definitions of body neutrality; rather, it reflects body positivity principles. Importantly, while some videos contained both #bodyneutrality and #bodypositivity hashtags (approximately one third), multiple videos solely containing the #bodyneutrality hashtag also showcased body positivity principles.

In sum, this theme provided insight into:

- the implicit or explicit overlap between the body neutrality and the body positivity movements as portrayed in the TikTok videos;
- the strive to distinguish the body neutrality movement from the body positivity movement;
- the preference for the body neutrality movement, presented as superior to the body positivity movement.

## Discussion

The current study examined how TikTok creators employ #bodyneutrality hashtags to construct meaning and generate discourse. In this way, our findings move beyond Pellizzer and Wade’s seminar work to facilitate an understanding of how body neutrality is understood and employed on social media [3]. Overall, we generated three themes to explain constructions of body neutrality on TikTok: (1) The normalisation of diverse bodies, (2) The rejection of appearance as fundamentally important, and (3) Body neutrality is (better than) body positivity.

The first of our themes – “the normalisation of diverse bodies” – spoke to how TikTok body neutrality content creators are working to validate diverse body types. In other words, rather than suggesting all bodies are beautiful (akin to body positivity), body neutrality content creators asserted that all bodies are good bodies (i.e., worthy, important, healthy) because the very experience of having a body is fundamentally normal, natural, and human. In this sense, creators expanded on existing body neutrality definitions. Pellizzer and Wade analysed and synthesised existing definitions of body neutrality across 107 websites and found that three core pillars underpin the movement: (1) body neutrality is more realistic and flexible than body positivity, (2) appreciating, respecting, and caring for the functionality of the body is paramount, and (3) self-worth is not defined by appearance [3]. Building on this, TikTok discourse seems to suggest that body neutrality perhaps has fourth and fifth pillars as well: (4) all bodies are normal, natural, and valid and (5) all types of bodies can be healthy bodies. Where existing online conceptualisations of body neutrality merely speak to reducing focus on appearance, TikTok discourse seems to additionally focus on increasing focus on normalisation.

The importance of normalizing different bodies and physical characteristics has been often described in the body image field in connection to social media use. An attempt to normalize different body types on social media can be traced back to Instagram and the “Instagram vs Reality” posts that were first popularised in the early 2010s. These posts featured two complementary photos of the poster uploaded side by side: one ideal “Instagram” photo (e.g., posed, edited) and one more realistic version with a more natural/neutral pose and without editing. These posts were found to be beneficial for body image [41]. Thus, it seems that TikTok representations of body neutrality in part draw inspiration from previous social media body image movements that also pushed to normalise diverse bodies.

Knowing that there is still demand for this kind of normalisation content online might inform future directions in policy and academic research. Namely, TikTok content suggests that a shift away from appearance should not mean ignoring appearance-based stigmatisation; rather, dismantling stigma is framed as paramount to achieving a body neutral society. Moreover, this finding emphasises how TikTok can be employed as a platform to foster critical thinking and social media literacy, including an understanding of how social media representations of appearance may be unrealistic. Emerging literature already underscores how seeing social media posts that tackle unrealistic beauty standards help individuals to foster functional relationships with the body [42]. Future research and policy should therefore continue to explore the power of TikTok body image movements to create social change.

Our second theme – “the rejection of appearance as fundamentally important” – speaks to existing body neutrality principles. As per Pellizzer and Wade’s framework, body neutrality TikTok content in the present study spoke to diminishing the importance we place on beauty [3]. It emphasised that an individual’s characteristics (e.g., interests, hobbies, personal taste) are more interesting than how they look and framed the body as a vessel through which to celebrate/actualise these characteristics. Coupled with a focus on normalising diverse body types (as per Theme 1), Theme 2’s rejection of beauty may explain (in part) why viewing #bodyneutrality content has a positive impact on body image [28, 29]. 

Moreover, this theme demonstrates the connection between TikTok constructions of body neutrality and the construct of positive body image [27]. Rejection of beauty standards and appreciation of other non-appearance-related features is a component of the broad conceptualization of positive body image. Ideally, someone with positive body image would appreciate their physical appearance regardless of their alignment with conventional beauty standards. Additionally, however, positive body image involves celebrating non-body-oriented (e.g., personality) characteristics and rejecting the idea that beauty is important [6]. Likewise, de-emphasizing the importance placed on appearance in favour of other qualities, such as personal interests, is a key component of psychological interventions aimed at improving body image [43]. Since body neutrality content promotes the same messages, it could be considered as aligned with known effective means of enhancing positive body image.

The last of our themes – “body neutrality is (better than) body positivity” – explored the similarities and differences between body neutrality and body positivity as understood by TikTok creators. It also examined why some creators felt more aligned with the body neutrality movement. We found that creators depicted similarities and differences between body neutrality and body positivity in line with existing definitions of the two movements (e.g., that both movements argue exercise ought to be for joy and celebration rather than a form of ‘punishment’; that body positivity is more appearance-centred than body neutrality). Similarities could be explained by both movements being underpinned by shared messages related to the construct of positive body image [27]. 

Interestingly, we also found that several creators framed body neutrality as superior to body positivity. In line with Pellizzer and Wade’s framework, most creators who compared the two movements praised body neutrality for being more realistic and flexible [3]. Existing literature has previously highlighted that body positivity content can be perceived as “toxic”, forcing people to share unrealistic love and appreciation for the physical appearance [3, 27]. Relatedly, the term body positivity is sometimes considered as having been appropriated for commercial purposes, and therefore is no longer considered an ‘authentic’ movement [27]. Thus, individuals interested in improving their relationships with their body may be leaning towards body neutrality as the more appropriate representation of a functional relationship with the body, harshly criticising the “corruption” of body positivity.

Because little existing literature has explored how body neutrality is understood and represented within social media discourse, these novel findings suggest that body neutrality does indeed resonate with people experiencing body image concerns and thus that body neutrality principles ought to be incorporated into treatment and intervention programs.

These findings corroborated how body neutrality content could be useful in promoting a positive attitude toward the body since it overlaps with principles related to positive body image [27]. Additionally, body neutrality could be considered as a mean to foster critical thinking to navigate social media, fostering social media literacy [44]. 

### Creator diversity and other considerations

Through the quantitative analysis of descriptive and demographic characteristics of #bodyneutrality content creators, we found that creators were mostly (perceived to be) young White women. This information is in line with the recent analysis conducted by Hallward et al. on body neutrality and body positivity content [1]. Interestingly, the majority of the TikTok creators were perceived to be in their 20s (20 to 29 years old), contrary to the study on #bodypositivity TikTok content, in which they were perceived as younger (i.e., 15 to 20 years old) [45]. This finding could suggest differences in the audience of these TikTok videos: even though both age groups are known to follow appearance-focused accounts on social media to a similar extent, it is possible that body positivity is more appealing to a younger audience (e.g., adolescents or late adolescents) while body neutrality speaks more to adult audiences [46]. This hypothesis could be further examined by contrasting hashtag engagement across various age groups. Differences in audiences may also reflect differences in how adolescents and young adults may strive to build a functional relationship with their bodies. Adolescents, especially girls, may feel the need to showcase appreciation and love for their body. The great importance placed on appearance, which characterize this age group, may be responsible of this process [47]. On the other hand, young adults may be characterized by diverse social responsibilities and interests beyond appearance [48]. Thus, normalizing diverse bodies and reducing emphasis on appearance could be more relevant. Future studies should investigate the frequency of consumption of body neutrality and positivity content in different age groups.

In general, this homogeneity in TikTok profiles could create a lack of cultural and gender representation and increase the marginalisation of minorities in the body neutrality movement [25]. It could also threaten the purposes of the body neutrality movement, such as “the normalisation of diverse bodies”, leading viewers to believe that only “White young female bodies” are normal.

All but three of the analysed TikTok videos portrayed at least a person. Thus, despite leaning towards removing emphasis on appearance, body neutrality content still relies on showcasing bodies. Implications on body image dimensions should be examined, comparing this content to content without human figures (e.g., TikTok videos displaying an environment accompanied with an audio recording).

Finally, one-third of the TikTok videos presented #bodypositivity in association with #bodyneutrality. This finding further corroborated the connection between body positivity and body neutrality in online discourse [1]. Moreover, it underscores the shared connection and meaning between body neutrality, body positivity, and the construct of positive body image.

### Limitations

The findings of this study need to be interpreted considering several limitations. First, we collected content at only one time-point. Given that social media discourse can change rapidly over time, our research cannot speak to the evolving understanding of body neutrality on TikTok. Second, our findings can only speak to the nature of TikTok discourse in an Australian context; it is unclear whether body neutrality is understood similarly on a global scale. Thirdly, we analysed only a limited number of Spanish and Italian videos. Thus, it is likely that Spanish and Italian #bodyneutrality content is characterised by unique features not identified in the present study. That said, evidence of non-English #bodyneutrality videos on TikTok suggest the diffusion of this concept in non-English First Language populations. This finding in and of itself is important. Finally, we did not account for the possibility that TikTok content could have been mostly from the same creators, limiting potentially the variability in the videos.

## Conclusion

Body neutrality is receiving a great deal of attention from both scholars and the general public, with body neutrality discourse rife across TikTok and other social media platforms. After analysing 178 TikTok videos across three languages, we developed three themes to summarise how body neutrality is currently represented on TikTok: (1) The normalisation of diverse bodies, (2) The rejection of appearance as fundamentally important, and (3) Body neutrality is (better than) body positivity. We determined that TikTok creators often expanded on existing definitions of body neutrality to incorporate destigmatisation and normalisation discourse. We also noted that creators emphasised the role body neutrality plays in healthy body image over and above (or in place of) body positivity principles. Overall, our findings went beyond existing explorations of online discourse to comprehensively examine how body neutrality is understood on social media.

## Data Availability

The TikTok URLs that were employed for the analysis of this study are available on the Open Science Framework (OSF; https://osf.io/kf8my/?view_only=bd71ad360689487b91ff10bf75146a81).

## References

[1] Hallward L, Feng O, Duncan LR. An exploration and comparison of #BodyPositivity and #BodyNeutrality content on TikTok. Eat Behav. 2023. 10.1016/j.eatbeh.2023.101760. 50;101760. 37329772 10.1016/j.eatbeh.2023.101760

[2] Smith AC, Ahuvia I, Ito S, Schleider JL. Project Body Neutrality: piloting a digital single-session intervention for adolescent body image and depression. Int J Eat Disord. 2023;56(8):1554–69. 10.1002/eat.23976. 37129116 10.1002/eat.23976 PMC10524309

[3] Pellizzer ML, Wade TD. Developing a definition of body neutrality and strategies for an intervention. Body Image. 2023;46:434–42. 10.1016/j.bodyim.2023.07.006. 37573765 10.1016/j.bodyim.2023.07.006

[4] Cash TF, Pruzinsky TP. Body images: development, deviance, and change. New York: Guilford Press; 1990.

[5] Grogan S. Body image and health: contemporary perspectives. J Health Psychol. 2006;11(4):523–30. 10.1177/135910530606501. 16769732 10.1177/1359105306065013

[6] Tylka TL, Wood Barcalow NL. What is and what is not positive body image? Conceptual foundations and construct definition. Body Image. 2015;14:118–29. 10.1016/j.bodyim.2015.04.001. 25921657 10.1016/j.bodyim.2015.04.001

[7] Thompson JK, Heinberg LJ, Altabe M, Tantleff-Dunn S. Exacting beauty: theory, assessment and treatment of body image disturbance. Washington, D.C.: American Psychological Association; 1999. 10.1037/10312-000.

[8] Roberts SR, Maheux AJ, Hunt RA, Ladd BA, Choukas-Bradley S. Incorporating social media and muscular ideal internalization into the tripartite influence model of body image: towards a modern understanding of adolescent girls’ body dissatisfaction. Body Image. 2022;41:239–47. 10.1016/j.bodyim.2022.03.002. 35306356 10.1016/j.bodyim.2022.03.002

[9] Tylka TL, Rodgers RF, Calogero RM, Thompson JK, Harriger JA. Integrating social media variables as predictors, mediators, and moderators within body image frameworks: potential mechanisms of action to consider in future research. Body Image. 2023;44:197–221. 10.1016/j.bodyim.2023.01.004. 36709634 10.1016/j.bodyim.2023.01.004

[10] Griffiths S, Castle D, Cunningham M, Murray SB, Bastian B, Barlow FK. How does exposure to thinspiration and fitspiration relate to symptom severity among individuals with eating disorders? Evaluation of a proposed model. Body Image. 2018;27:187–95. 10.1016/j.bodyim.2018.10.002. 30359868 10.1016/j.bodyim.2018.10.002

[11] Zhang J, Wang Y, Li Q, Wu C. The relationship between SNS usage and disordered eating behaviors: a Meta-analysis. Front Psychol. 2021. 10.3389/fpsyg.2021.641919. 12;641919. 34413807 10.3389/fpsyg.2021.641919 PMC8367749

[12] Barron AM, Krumrei-Mancuso EJ, Harriger JA. The effects of fitspiration and self-compassion Instagram posts on body image and self-compassion in men and women. Body Image. 2021;37:14–27. 10.1016/j.bodyim.2021.01.003. 33556914 10.1016/j.bodyim.2021.01.003

[13] Pryde S, Prichard I. TikTok on the clock but the #fitspo don’t stop: the impact of TikTok fitspiration videos on women’s body image concerns. Body Image. 2022;43:244–52. 10.1016/j.bodyim.2022.09.004. 36194987 10.1016/j.bodyim.2022.09.004

[14] Stevens A, Griffiths S. Body positivity (#BoPo) in everyday life: an ecological momentary assessment study showing potential benefits to individuals’ body image and emotional wellbeing. Body Image. 2020;35:181–91. 10.1016/j.bodyim.2020.09.003. 33068893 10.1016/j.bodyim.2020.09.003

[15] Yee ZW, Griffiths S, Fuller-Tyszkiewicz M, Blake K, Richardson B, Krug I. The differential impact of viewing fitspiration and thinspiration images on men’s body image concerns: an experimental ecological momentary assessment study. Body Image. 2020;35:96–107. 10.1016/j.bodyim.2020.08.008. 32977202 10.1016/j.bodyim.2020.08.008

[16] Cohen R, Fardouly J, Newton-John T, Slater A. #BoPo on Instagram: an experimental investigation of the effects of viewing body positive content on young women’s mood and body image. New Media Soc. 2019;21(7):1546–64. 10.1177/1461444819826530.

[17] Dhadly PK, Kinnear A, Bodell LP, #BoPo. Does viewing body positive TikTok content improve body satisfaction and mood? Eat Behav. 2023. 10.1016/j.eatbeh.2023.101747. 50;101747. 37263141 10.1016/j.eatbeh.2023.101747

[18] Fioravanti G, Svicher A, Ceragioli G, Bruni V, Casale S. Examining the impact of daily exposure to body-positive and fitspiration Instagram content on young women’s mood and body image: an intensive longitudinal study. New Media Soc. 2023;25(12):3266–88. 10.1177/14614448211038904.

[19] Kvardova N, Machackova H, Smahel D. A moderated mediation model for body-positive online content and body image among adolescents. Body Image. 2022;42:370–4. 10.1016/j.bodyim.2022.07.002. 35930872 10.1016/j.bodyim.2022.07.002

[20] Mancin P, Cerea S, Bottesi G, Ghisi M. Instagram use and negative and positive body image: the relationship with following accounts and content and filter use among female students. Curr Psychol. 2024;43:10669–81. 10.1007/s12144-023-05204-w.

[21] Mink D, Szymanski DM. TikTok use and body dissatisfaction: examining direct, indirect, and moderated relations. Body Image. 2022;43:205–16. 10.1016/j.bodyim.2022.09.006. 36191378 10.1016/j.bodyim.2022.09.006

[22] Nelson SL, Harriger JA, Miller-Perrin C, Rouse SV. The effects of body-positive Instagram posts on body image in adult women. Body Image. 2022;42:338–46. 10.1016/j.bodyim.2022.07.013. 35926363 10.1016/j.bodyim.2022.07.013

[23] Schettino G, Capasso M, Caso D. The dark side of #bodypositivity: the relationships between sexualized body-positive selfies on Instagram and acceptance of cosmetic surgery among women. Comput Hum Behav. 2023. 10.1016/j.chb.2022.107586. 140;107586.

[24] Vendemia MA, DeAndrea DC, Brathwaite KN. Objectifying the body positive movement: the effects of sexualizing and digitally modifying body-positive images on Instagram. Body Image. 2021;38:137–47. 10.1016/j.bodyim.2021.03.017. 33887562 10.1016/j.bodyim.2021.03.017

[25] Cohen R, Irwin L, Newton-John T, Slater A, #bodypositivity. A content analysis of body positive accounts on Instagram. Body Image. 2019;29:47–57. 10.1016/j.bodyim.2019.02.007. 30831334 10.1016/j.bodyim.2019.02.007

[26] Lazuka RF, Wick MR, Keel PK, Harriger JA. Are we there yet? Progress in depicting diverse images of beauty in Instagram’s body positivity movement. Body Image. 2020;34:85–93. 10.1016/j.bodyim.2020.05.001. 32534269 10.1016/j.bodyim.2020.05.001

[27] Wood-Barcalow NL, Alleva JM, Tylka TL. Revisiting positive body image to demonstrate how body neutrality is not new. Body Image. 2024;50:101741. 10.1016/j.bodyim.2024.101741. 38850714 10.1016/j.bodyim.2024.101741

[28] Brathwaite KN, DeAndrea DC, Vendemia MA. Non-sexualized images and body-neutral messaging Foster Body Positivity Online. Soc Media Soc. 2023;9(4). 10.1177/20563051231207852.

[29] Seekis V, Lawrence RK. How exposure to body neutrality content on TikTok affects young women’s body image and mood. Body Image. 2023;47. 10.1016/j.bodyim.2023.101629. 10.1016/j.bodyim.2023.101629 37742535

[30] Statista S. 2023 [cited 2023 Sept 29]. https://www.statista.com/forecasts/1142687/tiktok-users-worldwide

[31] Dhir A, Tsai CC. Understanding the relationship between intensity and gratifications of Facebook use among adolescents and young adults. Telemat Inf. 2017;34(4):350–64. 10.1016/j.tele.2016.08.017.

[32] Rodgers RF, McLean SA, Gordon CS, Slater A, Marques MD, Jarman HK, Paxton SJ. Development and validation of the motivations for Social Media Use Scale (MSMU) among adolescents. Adolesc Res Rev. 2021;6:425–35. 10.1007/s40894-020-00139-w.

[33] Kneeland J. Why Body Neutrality Works Better Than Body Positivity [Internet]. TIME; 2023 [cited 2023]. https://time.com/6279423/body-positivity-vs-neutrality/

[34] Braun V, Clarke V. Using thematic analysis in psychology. Qual Res Psychol. 2006;3(2):77–101. 10.1191/1478088706qp063oa.

[35] Herrick SSC, Hallward L, Duncan LR. This is just how I cope: an inductive thematic analysis of eating disorder recovery content created and shared on TikTok using #EDrecovery. Int J Eat Disord. 2021;54(4):516–26. 10.1002/eat.23463. 33382136 10.1002/eat.23463

[36] Minadeo M, Pope L. Weight-normative messaging predominates on TikTok-A qualitative content analysis. PLoS ONE. 2022;17(11):e0267997. 10.1371/journal.pone.0267997. 36318532 10.1371/journal.pone.0267997 PMC9624392

[37] Tiggemann M, Zaccardo M. Strong is the new skinny’: a content analysis of #fitspiration images on Instagram. J Health Psychol. 2018;23(8):1003–11. 10.1177/1359105316639436. 27611630 10.1177/1359105316639436

[38] Boepple L, Ata RN, Rum R, Thompson JK. Strong is the new skinny: a content analysis of fitspiration websites. Body Image. 2016;17:132–5. 10.1016/j.bodyim.2016.03.001. 27045871 10.1016/j.bodyim.2016.03.001

[39] McHugh ML. Interrater reliability: the kappa statistic. Biochem Med (Zagreb). 2012;22(3):276–82. 23092060 PMC3900052

[40] Byrne D. A worked example of Braun and Clarke’s approach to reflexive thematic analysis. Qual Quant. 2022;56:1391–412. 10.1007/s11135-021-01182-y.

[41] Tiggemann M, Anderberg I. Social media is not real: the effect of ‘Instagram vs reality’ images on women’s social comparison and body image. New Media Soc. 2020;22(12):2183–99. 10.1177/1461444819888720.

[42] Paxton SJ, McLean SA, Rodgers RF. My critical filter buffers your app filter: social media literacy as a protective factor for body image. Body Image. 2022;40:158–64. 10.1016/j.bodyim.2021.12.009. 34968853 10.1016/j.bodyim.2021.12.009

[43] Alleva JM, Sheeran P, Webb TL, Martijn C, Miles E. A Meta-Analytic review of stand-alone interventions to improve body image. PLoS ONE. 2015;10(9):e0139177. 10.1371/journal.pone.0139177. 26418470 10.1371/journal.pone.0139177 PMC4587797

[44] Rodgers RF, McLean SA, Paxton SJ. Enhancing understanding of social media literacy to better inform prevention of body image and eating disorders. Eat Disord. 2024;1–19. 10.1080/10640266.2024.2336700. 10.1080/10640266.2024.2336700 38590160

[45] Harriger JA, Wick MR, Sherline CM, Kunz AL. The body positivity movement is not all that positive on TikTok: A content analysis of body positive TikTok videos. Body Image. 2023;46:256–64. 10.1016/j.bodyim.2023.06.003. 37379612 10.1016/j.bodyim.2023.06.003

[46] Vall-Roqué H, Andrés A, Saldaña C. The impact of COVID-19 lockdown on social network sites use, body image disturbances and self-esteem among adolescent and young women. Prog Neuropsychopharmacol Biol Psychiatry. 2021. 10.1016/j.pnpbp.2021.110293. 110;110293. 33662532 10.1016/j.pnpbp.2021.110293 PMC8569938

[47] Micali N, Ploubidis G, De Stavola B, Simonoff E, Treasure J. Frequency and patterns of eating disorder symptoms in early adolescence. J Adolesc Health. 2014;54(5):574–81. 10.1016/j.jadohealth.2013.10.200. 24360247 10.1016/j.jadohealth.2013.10.200

[48] Gattario KH, Frisén A. From negative to positive body image: men’s and women’s journeys from early adolescence to emerging adulthood. Body Image. 2019;28:53–65. 10.1016/j.bodyim.2018.12.002. 30583277 10.1016/j.bodyim.2018.12.002

